# Lifestyle and Psychological Factors Affecting Eating Habits and Physical Activity Among Government Servants in the States With the Highest Cumulative Cases in Malaysia During the COVID-19 Pandemic

**DOI:** 10.3389/fpubh.2022.816530

**Published:** 2022-04-19

**Authors:** Nur Hana Hamzaid, Zeesha Gloria Rayner Gumisi, Syaidatul Khafizah Ahmad Helme, Norhazirah Azmi, Mohd. Razif Shahril

**Affiliations:** ^1^Faculty of Health Sciences, Centre for Rehabilitation and Special Needs Studies (iCaRehab), Universiti Kebangsaan Malaysia, Kuala Lumpur, Malaysia; ^2^Department of Dietetics, Faculty of Health Sciences, Universiti Kebangsaan Malaysia, Kuala Lumpur, Malaysia; ^3^Department of Dietetics and Food Service, Children Specialist Hospital, Universiti Kebangsaan Malaysia, Kuala Lumpur, Malaysia; ^4^Faculty of Health Sciences, Centre for Healthy Ageing and Wellness (H-CARE), Universiti Kebangsaan Malaysia, Kuala Lumpur, Malaysia

**Keywords:** COVID-19, lifestyle, eating habits, psychological factors, physical activity, workers

## Abstract

**Introduction:**

Like many other countries, the federal government of Malaysia took the initiative to implement nationwide home confinement as a preventive measure in response to the pandemic COVID-19 outbreak, better known as Movement Control Order (MCO). Many have suffered economically, which led to poor states of well-being. This study investigates the relationship between lifestyle, psychological factors affecting eating habits, and physical activity among government servants in states with the highest cumulative cases during the COVID-19 pandemic.

**Methods:**

A cross-sectional online survey was conducted among 210 government servants from four states (Selangor, Sabah, Kuala Lumpur, and Johor) from May 2021 to June 2021. Three validated questionnaires were used, namely, Emotional-Eater Questionnaire (EEQ), and Malay International Physical Activity Questionnaire (IPAQ-M) from López-Moreno et al. (1).

**Results:**

There were significant gender differences (*P* < 0.001) for supplement intake, with 30.4% among men and 62.3% in women. Almost half (49.1%) were classified as emotional or very emotional eaters. Also, a significant difference (*P* < 0.005) was found in the perception of boredom and apathy between men (35.7%) and women (31.8%). A majority (56.2%) stated that their mood positively affected their eating habits. The total metabolic equivalent of task (MET) for 132 subjects was 3495.8 ± 3862.7 min/week for physical activity. Significant differences were observed between MET and gender (Mann–Whitney *U*-test: *P* = 0.019), with men showing higher MET value (5001.4 ± 5354.0 min/week men, 2864.3 ± 2754.3 min/week women). A significant difference was observed among women for body weight and body mass index (BMI) before and during MCO (*P* < 0.001). For the emotional eater, there were correlations with eating habits before the MCO for quantity of food intake (*P* = 0.003), frequency of mealtime *(P* < 0.001), changes of food habits (*P* = 0.005), cooking methods (*P* = 0.016), and frequency of food intake (*P* = 0.020). There is no correlation (*P* = 0.577) between psychological factors affecting eating habits and physical activity during COVID-19.

**Conclusion:**

Changes were reported before and during MCO on lifestyle, eating habits, and physical activity. Such information will help design strategies to improve the economic and health status among government servants in Malaysia during the implementation of MCO or something similar.

## Introduction

The rapid spread of COVID-19 continues to substantially impact the economy and well-being of people worldwide. The year 2020 began with the spread of the novel coronavirus outbreak that causes severe acute respiratory syndrome coronavirus 2 (SARS-COV-2) which was named COVID-19 by the World Health Organization (WHO) (2). In January 2020, the WHO has declared the outbreak a Public Health Emergency of International Concern and a Global Pandemic in March 2020.

The infection and spread of the disease at high rates have alarmed health agencies and citizens. The first local case of COVID-19 detected in Malaysia was on January 23, 2020, involving two citizens from Sabah and one from Selangor. The government took a preventive measure to combat the COVID-19 outbreak through social and physical distancing by implementing a nationwide Movement Control Order (MCO) starting March 18, 2020 with the recommendation of the WHO. The MCO was a directive closure of all government and private premises except for essential services which included water, electricity, energy, telecommunications, postal, transportation, irrigation, oil, gas, fuel, lubricants, broadcasting, financial, banking, health, pharmacy, fire, prison, port, airport, security, defense, cleaning, retail, and food supply. In addition, the closing of all nurseries, government, private schools, and public and private higher education institutions was required. A significant increase in cases was seen in October 2020, which caused the Conditional Movement Control Order (CMCO) and the Enhanced Movement Control Order (EMCO) to be implemented in selected states in Malaysia. The social and physical distance practices were crucial in combating the COVID-19 outbreak. However, it has also altered many aspects of life, including lifestyle, psychological factors affecting eating habits, and physical activity during the pandemic (3).

Quarantine or MCO is associated with restriction of movement. It includes daily work routines, which can cause emotional changes such as anxiety or boredom. Changes in eating habits have been reported and associated with greater energy and macronutrient intake during the COVID-19 pandemic (1). As humans are inherently social, long periods of social distancing may put psychological stress which can lead to a higher intake of larger quantities of food to cope with fear and anxiety (4).

The COVID-19 outbreak has also impacted lifestyle and daily work routine. Various studies on lifestyle changes during COVID-19 were conducted, including eating habits, supplementation, smoking habits, alcohol intake, and sleeping patterns. Increased time at home has led to excessive consumption of food which were related to social, emotion, or food cravings, commonly known as “comfort food” to help in reducing stress. “Comfort food” is normally high in simple carbohydrates which aid to reduce stress by inducing high serotonin production, thus positively impacts one's emotions. However, the effect on “comfort foods” is directly proportional to the glycemic index of food, which is closely related to increased risk of obesity, diabetes, and cardiovascular disease, all of which are known to increase risk of complications among COVID-19 victims (5). In addition, psychological factors such as anxiety, loneliness, and boredom have also affected eating habits during the COVID-19 pandemic. The isolation has caused great pressure, resulting in high intake of food. In addition to that, news and information on COVID-19 in social media can be stressful and overwhelming, leading to excessive eating (1).

Increased time spent at home during the COVID-19 has influenced various changes in smoking habits. The study from Di Renzo et al. (5) reported that there was an increment in smoking habits among Italians, while Sidor et al. (6) reported that there were no changes in smoking habits among Polish people. In addition, changes in sleeping patterns during the pandemic has also affected individuals' quality of sleep. A study in the Middle East and North Africa (MENA) reported a decline in sleep quality during the COVID-19 pandemic (7). However, a study in Portugal by Antunes et al. (8) reported that the sleep quality of Portuguese had no changes. An increment in consumption of dietary supplements like Vitamin C and D was seen during the COVID-19 pandemic as an effort to increase immunity. Furthermore, a significant change in the consumption of alcohol caused by increased time spent at home and stress was observed during the pandemic.

The new norm has also altered physical activity during the outbreak. A previous study done by López-Moreno et al. (1) in Spain reported that 44.7% of respondents did not perform any exercises or physical activities during the pandemic due to the limited social and physical distance practiced. In addition, prolonged screen exposure to smartphones, computers, and televisions has contributed to sedentary behavior together with experiencing barriers in doing exercise that resulted from the closure of the recreational parks and sports centers during. This has consequently led to a decline in physical activity level. Additionally, Work from Home Policy (WFH) has contributed to the reduction of movement compared to before the pandemic (9).

## Materials and Methods

### Study Design and Population

This was a cross-sectional study conducted online among Malaysian government servants. Particularly, men and women aged ≥18 years from four states with the highest cumulative COVID-19 cases in Malaysia (Selangor, Sabah, Johor, and Kuala Lumpur). Data were collected from May to June 2021 to assess the lifestyle behaviors, psychological factors affecting eating habits, and physical activity among government servants during the COVID-19 outbreak in Malaysia. Participants were mainly recruited through social media (Facebook, WhatsApp, Telegram, Instagram, and email) and were asked to complete an online questionnaire using the Google Forms web survey platform. A total of 210 participants were recruited, including 56 men and 154 women.

### Ethical Aspects

A brief description of the study and its purpose and an informed consent form was provided at the beginning of the electronic page containing the invitation for survey participation. All subjects were considered to have given their consent after clicking the “accept” icon, indicating that they have understood the informed consent requirements. This study has received approval from the Research and Ethics Committee of The National University of Malaysia (UKM) with ethical code: UKM PPI/111/8/JEP-2021-269.

### Assessment of Socio-Demographic, Anthropometric, Lifestyle, and Psychological Factors Affecting Eating Habits and Physical Activity Level

Data collection was carried out through a digital self-administered electronic questionnaire using Google Form. The instrument was designed and culturally adapted to be applied in the studied country. It included questions on sociodemographic, anthropometric, lifestyle changes, psychological factors affecting eating habits, and physical activity during the COVID-19 pandemic. The following sociodemographic characteristics were evaluated: age in years; state of living during home confinement (Selangor, Sabah, Kuala Lumpur, Johor); occupation; gender (male or female); educational level (secondary school, vocational education, Foundation/Matriculation, Diploma, Bachelor's degree, Masters, Ph.D.); living status during MCO (living alone, with one person, two persons, three persons, four persons, or more); and whether they are working from home (yes or no). The anthropometric data were collected in Body Mass Index (BMI) (body weight; before and during MCO in kg divided by height in meters squared). A set of questionnaires from López-Moreno et al. (1) were adopted to assess the lifestyle during COVID-19. It included the protection participants used during lockdown (face mask, gloves, and face shield), food habits (increased food consumption, specific food consumed, snacking, cooking practices during MCO, daily number of consumed meals and frequency of meals before and during MCO, sources of food during MCO, three most consumed foods, and soft drinks and type consumed), frequency and type of alcohol consumption, smoking frequency during MCO, dietary supplements consumption during MCO, and sleep quality and exercise before and during MCO with respect to the time and intensity dedicated.

Phychological factors, namely, mood changes during lockdown were evaluated using the Emotional Eater Questionnaire (EEQ) by Garaulet et al. (10), with respect to feeding, nervousness, sleep-problem, and overall feeling about life-affecting eating habits. The EEQ is a 10-item questionnaire developed to assess the extent to which emotions affect eating behavior. The question had four possible replies: (1) never, (2) sometimes, (3) generally, and (4) always. The total score ranged from 0 to 30. The subjects were classified into 4 groups: non-emotional eater (score 0–5), low emotional eater (score 6–10), emotional eater (score 11–20), and very emotional eater (score 21–30). The self-administered 7-day activity questionnaire, the Malay International Physical Activity Questionnaire (IPAQ-M), that was validated for Malaysian population (11) was used in this study. The questions included were total frequency and duration of vigorous activity, moderate activity, walking, and sitting. The following metabolic equivalent of task (MET) values were used: walking = 3.3 METs, moderate activity = 4.0 METs, and vigorous activity = 8.0 METs. The MET-minutes per week (MET-min/week) was calculated as follows: minutes of activity/day × days per week × MET level. The total amount of physical activity scores obtained from the IPAQ-M was classified into three categories, namely, low, moderate, or high physical activity level, according to the scoring protocol on the IPAQ Web site guidelines (11).

### Data Analysis

Data were cleaned for duplicates before importing and analyzing using SPSS 22. Descriptive statistics of the socio-demographic characteristics of the participants were then conducted. EEQ was based on a 30-point scoring system using the cut-offs (Non-emotional eater: ≤5; Low emotional eater: 6–10; Emotional eater: 11–20; Very emotional eater: 21–30) used by Garaulet et al. (10). The International Physical Activity Questionnaire (IPAQ) was based on MET values (walking = 3.3 METs, moderate intensity = 4.0 METs, vigorous intensity = 8.0 METs) with the following calculation for total MET-minutes/week: Walk (METs × min × days) + Moderate Intensity (METs × min × days) + Vigorous Intensity (METs × min × days). Data were presented as mean ± SD and percentages in parentheses (%) for categorical variables. Paired *T*-test was conducted to determine the difference between the bodyweight before MCO and the body weight during MCO of the same participant. Independent *t*-test was used to determine the significant difference between the means in the MET value (min/week) and the gender of the participants. Chi-Square test was performed to evaluate the association of lifestyle, emotional eater classification (non-emotional eater, low emotional eater, emotional eater, and very emotional eater), physical activity level (low, moderate, and high), differences in body weight, and BMI before and during MCO with gender of the participants. Next, the Chi-Square test was also performed to evaluate the association of lifestyle and influence of psychological status (non-emotional eater, low emotional eater, emotional eater, and very emotional eater). Chi-Square test was also conducted to evaluate the association of influence of psychological status (non-emotional eater, low emotional eater, emotional eater, and very emotional eater) and physical activity level during MCO (low, moderate, and high). Results were significant at *P* < 0.05 for all analyses.

## Results

### Sociodemographic Characteristics

The studied sample consisted of 210 respondents, with the majority of them being women (73.3%). The mean age of participants was 41.2 years, and the age range was 22 to 59 years. The largest group was from Sabah (45.7%), followed by Selangor (32.4%), Kuala Lumpur (11.4%), and Johor (10.5%). The majority of participants (50%) hold bachelor's degrees. Also, 68.1% of participants live with four or more household members, and 82.5% of participants have been working from home during MCO (Table 1).

**Table 1:** Sociodemographic characteristics of respondents (*n* = 210).

		***n***	**Percentage (%)**
Gender	Male	56	26.7
	Female	154	73.3
State	Selangor	69	32.4
	Sabah	97	45.7
	Kuala Lumpur	24	11.4
	Johor	22	10.4
Educational level	Secondary school	22	11.0
	Foundation/Matriculation	3	1.4
	Diploma	26	12.4
	Degree	105	50.0
	Master	39	17.6
	PhD	16	7.1
Household	Living alone	1	3.3
	2 people	24	11.9
	3 people	36	16.7
	4 or more people	145	68.1
Work from home	Yes	174	82.5
	No	37	17.5

### Lifestyle Behaviors

In general, 62.4% of the participants reported changing their eating habits during the home confinement. A positive change can be seen as most participants reported improved eating behavior which was evaluated through eating patterns at home, reuse of surplus food, shopping list planning, menu planning, healthy food choices, and fresh food consumption during MCO. However, analyses on dietary intake showed a similar pattern between before and during MCO, with 46.2 and 40.5% answering no changes for the quantity of food intake and frequency of mealtime, respectively. The same was observed for the number of meals, wherein there was no change during MCO as the majority of the subjects declared eating three meals per day both before (39.0%) and during (35.7%) the home confinement. No statistical significance was also found between the number of meals consumption and gender (*P*-value before MCO = 0.158, *P*-value during MCO = 0.239).

Results for food choices show that 42.9% of the participants did not consume soft drinks during the home confinement. Among those who reported otherwise, carbonated drinks were the most preferred, followed by packet drinks (chrysanthemum tea, lemon tea, and soya) and cordials. In addition, most of the respondents reported carbohydrate food sources (rice, noodles, bread, biscuits, flour, and fruits) as the most essential food during MCO. At the same time, deep frying was the most preferred cooking method (65.7%).

Regarding the intake of supplements, the majority of subjects reported consuming supplements during MCO (53.8%), with a higher incidence among women (62.3%; *P* < 0.001; **Table 4**). The most frequently taken supplements were vitamin C, multivitamins, and fish oil. A small number of subjects, 7.6 and 5.7%, respectively consumed alcohol and tobacco during home confinement (Table 2), with beer, red wine, and traditional rice wine as the most consumed alcoholic beverages.

**Table 2:** Percentages of supplement intake, alcohol intake and tobacco intake, and sleep quality during MCO.

	**All (*n* = 210)**	**Men (*n* = 56)**	**Women (*n* = 154)**	***P-*value**
**Supplement intake**
Yes	113 (53.8%)	17 (30.4%)	96 (62.3%)	<0.001
No	97 (46.2%)	39 (69.6%)	58 (37.7%)	
**Alcohol intake**
Yes	16 (7.6%)	12 (21.4%)	4 (2.6%)	–
No	194 (92.2%)	44 (78.6%)	150 (97.4%)	
**Tobacco intake**
Yes	12 (5.7%)	11 (19.6%)	1 (0.6%)	–
No	198 (94.3)	45 (80.4%)	153 (99.4%)	
**Sleep quality**
Better	70 (33.3%)	21 (37.5%)	49 (31.8%)	0.100
Worse	27 (12.9%)	6 (10.7%)	21 (13.6%)	
Same	113 (53.8%)	29 (51.8%)	84 (54.5%)	

With regards to sleeping patterns, the average hours of sleep during MCO were 6.67 ± 1.353 h per day, higher than before MCO (6.30 ± 1.146 h). Statistical significance was found between sleeping hours before and during the home confinement (*P* < 0.001). An increment in the total number of subjects with more than 7 h of sleep per day was observed during home confinement (49.5%) compared to prior home confinement (31.4%). A total of 53.8% of participants stated that their sleep quality did not change before and during the pandemic. In addition, a similar finding was found between men and women (*P* > 0.005).

### Psychological Factors Affecting Eating Habits

During the MCO period, 43.8% of the participants reported to have not experienced any difficulty in falling asleep, 26.1% declared that they were looking for meaning in life, and 25.7% reported nervousness and distress (Table 3). While there were no differences in psychological factors between genders (*P* > 0.05), an exception was found for the perception of boredom and apathy, where a higher proportion of men answered yes compared to women (*P* = 0.029).

**Table 3:** Changes in emotional states during COVID-19.

**Variable**	**All (*n* = 210)**	**Men (*n* = 56)**	**Women (*n* = 154)**	***P*-value**
Nervousness/anxiety				
Yes	54 (25.7%)	10 (17.9%)	44 (28.5%)	0.259
No	67 (31.9%)	17 (30.2%)	50 (32.4%)	
Neutral	89 (42.4%)	29 (51.8%)	60 (39.0%)	
Boredom/apathy irritability				
Yes	69 (32.8%)	20 (35.7%)	49 (31.8%)	0.029
No	69 (32.8%)	17 (30.3%)	52 (33.7%)	
Neutral	72 (34.3%)	19 (33.9%)	53 (34.4%)	
Difficulty in falling asleep				
Yes	32 (15.2%)	11 (19.7%)	21 (13.6%)	0.657
No	92 (43.8%)	21 (37.5%)	71 (46.1%)	
Neutral	86 (41.0%)	24 (42.9%)	62 (40.3%)	
Looking for the meaning of life				
Yes	55 (26.1%)	14 (25.0%)	41 (26.6%)	0.792
No	65 (30.9%)	15 (26.8%)	50 (32.4%)	
Neutral	90 (42.9%)	27 (48.2%)	63 (40.3%)	
Think there is a relationship between food and health	210 (100%)	56 (100%)	154 (100%)	–
Likes to eat	203 (96.7%)	54 (96.4%)	149 (96.8%)	0.908
Consider that state of mind during confinement has influenced diet				
In a positive way	118 (56.2%)	30 (53.6%)	88 (57.1%)	0.100
In a negative way	28 (13.3%)	12 (21.4%)	16 (10.4%)	
EEQ				
Non-emotional eater	30 (14.3%)	9 (16.1%)	21 (13.6%)	0.306
Low-emotional eater	77 (36.7%)	15 (26.8%)	62 (40.3%)	
Emotional eater and very emotional eater	103 (49.1%)	32 (57.2%)	71 (46.1%)	

The findings also show that all participants perceived a relationship between food and health, and the majority (56.2%) of them stated that their mood positively affected their eating habits. When looking into the categories of emotional eating, most participants (49.1%) were classified as emotional eaters or very emotional eaters. In addition, the EEQ level was associated with quantity of food intake (*P* = 0.003) and frequency of mealtime (*P* < 0.001). However, there are no relationships between eating patterns at home (*P* = 0.175), reuse of excess food (*P* = 0.156), shopping list planning (*P* = 0.766), menu planning (*P* = 0.624), healthy food selection (*P* = 0.730), label reading (*P* = 0.772), snack purchase (*P* = 0.434), fresh food intake (*P* = 0.629), and processed/fast food intake (*P* = 0.076) and emotional level during MCO (*P* > 0.05).

It was found that emotional and very emotional eaters frequently used the frying method (73.8%) compared to non-emotional and low-emotional eaters who preferred healthier cooking methods (42.1%) during MCO (*P* = 0.016). However, there is no relationship between the consumption of soft drinks, smoking habits, alcohol intake, and emotional levels reported during MCO reported (*P* > 0.05) (Table 4).

**Table 4:** Association between psychological factors affecting eating habits and level of emotional eater (EEQ).

**Variables**	**Non-emotional and low emotional eater**	**Emotional and very emotional eater**	***P*-value**
**Cooking method**			
Frying	62 (57.9%)	76 (73.8%)	0.016
Grill, boiled and steam	45 (42.1%)	27 (26.2%)	
**Soft drinks intake**			
Yes	58 (54.2%)	62 (60.2%)	0.381
No	49 (45.8%)	41 (39.8%)	
**Changes in eating habits**			
Yes	57 (53.3%)	74 (71.8%)	0.005
No	50 (46.7%)	29 (28.2%)	
**Frequency of food intake before MCO**			
<3 times	25 (23.4%)	23 (22.3%)	0.020
3 times	48 (44.9%)	34 (33.0%)	
4 times	26 (24.3%)	23 (22.3%)	
5–6 times	8 (7.5%)	23 (22.3%)	
**Frequency of food intake during MCO**			
<3 times	24 (22.4%)	21 (20.4%)	0.170
3 times	42 (39.3%)	33 (32.0%)	
4 times	30 (28.0%)	27 (26.2%)	
5–6 times	11 (10.3%)	22 (21.4%)	
**Smoking habits**			
Yes	6 (5.6%)	6 (6.4%)	0.946
No	101 (94.4%)	97 (94.2%)	
**Alcohol intake**			
Yes	9 (8.4%)	7 (6.8%)	0.659
No	98 (91.6%)	96 (93.2%)	
**Variables**	**Non-emotional eater**	**Low-emotional eater**	**Emotional eater and very emotional eater**	***P*** **-value**
**Supplement intake**
Yes	12 (40.0%)	47 (61.0%)	54 (52.4%)	0.135
No	18 (60.0%)	30 (39.0%)	49 (47.6%)	
**Quality of sleep**
Better	9 (30.0%)	26 (33.8%)	35 (34.0%)	0.916
Same/worse	21 (70.0%)	51 (66.2%)	68 (66.0%)	

In addition, it was also found that there is a relationship between changes in eating habits and emotional levels. Particularly, higher percentage (71.8%) of emotional and very emotional eaters changed their eating habits during MCO (*P* = 0.005). Furthermore, there is also a relationship between the frequency of food intake before MCO and emotional levels (*P* = 0.020). Particularly, the intake of three meals contributed to the higher percentage (44.9%) of non-emotional and low-emotional eaters. However, there is no relationship between the frequency of food intake, supplement intake, and quality of sleep during MCO and the reported emotional levels (*P* > 0.05) (Table 4).

### Physical Activity Level During MCO

Most participants performed exercise during MCO. Specifically, 21.9% continued with the same training patterns they had before MCO, while 17.6% performed lower intensity exercise and 11.9% performed shorter periods of exercise compared to what they usually did before MCO. Meanwhile, 11.9% of the participants only started doing exercise during the home confinement. Moreover, the majority of participants reported an increase in the total time of doing house chores (54.3%) during MCO.

Based on IPAQ-M, a higher MET was observed in men (5001.4 ± 5354.0 min/week) compared to women (2864.3 ± 2754.3 min/week). Almost half of the subjects were categorized with high MET (45.5%) (Table 5). There was no association found between psychological factors affecting eating habits with MET categories (*P* = 0.577). However, most subjects with non-emotional and low emotional eaters had high MET (50.0%) during MCO (Table 6).

**Table 5:** Total MET and MET categories among government workers during MCO.

	**All (*n* = 132)**	**Men (*n* = 39)**	**Women (*n* = 93)**	***P-*value**
**MET (min/week)**	3495.8 (SD 3862.7)	5001.4 (SD 5354.0)	2864.3 (SD 2754.3)	0.019
**MET categories**				
Low	24 (18.2%)	3 (7.7%)	21 (22.6%)	–
Moderate	48 (36.4%)	13 (33.3%)	35 (37.6%)	
High	60 (45.5%)	23 (59.0%)	37 (39.8%)	

**Table 6:** Association between psychological factors affecting eating habits and MET categories among government servants during MCO.

	**Non-emotional and low emotional eater**	**Emotional and very emotional eater**	***P-*value**
Low	11 (16.7%)	13 (19.7%)	0.577
Moderate	22 (33.3%)	26 (39.4%)	
High	33 (50.0%)	27 (40.9%)	

### Changes in Weight Before and During MCO

Table 7 shows the changes in weight and BMI before and during MCO. Participants reported a significant decrease in body weight and BMI during MCO compared to prior to MCO (*P* < 0.001). Among women, there were also significant differences reported in body weight (*P* < 0.001) and BMI (*P* < 0.001) during MCO compared to before MCO.

**Table 7:** Weight changes and BMI before and during MCO among government servants during MCO.

	**All (*****n*** **=** **210)**			**Men (*****n*** **=** **56)**			**Women (*****n*** **=** **154)**		
	**Before**	**During**	***P-*value**	**Before**	**During**	***P-*value**	**Before**	**During**	***P-*value**
Weight (kg)	69.5 (SD 15.2)	67.2 (SD 14.5)	<0.001	77.2 (SD 13.5)	76.2 (SD 14.0)	0.189	65.3 (SD 14.6)	63.9 (SD 13.3)	<0.001
Height (m)	1.59 (SD 0.08)			1.67 (SD 0.06)			1.56 (SD 0.07)		
BMI (kg/m^2^)	27.1 (SD 5.5)	26.6 (SD 5.2)	<0.001	27.7 (SD 4.5)	27.4 (SD 4.7)	0.178	26.9 (SD 5.8)	26.3 (SD 5.3)	<0.001

## Discussion

This study had one main purpose: to assess the lifestyle behaviors, psychological factors affecting eating habits, and physical activity among government servants during the COVID-19 outbreak in Malaysia. Most countries implemented a quarantine period to deplete the positive cases of COVID-19, which included the MCO through the practice of physical distancing. Nevertheless, fear of the disease and the current increasing death cases of COVID-19 patients, along with the restrictions of one's freedom has worsened stress levels. Hence, producing alteration of habitual behaviors (5). The majority of the global population faced challenges upon the current trend of the COVID-19 pandemic, causing changes in lifestyle behaviors (2). MCO caused by the COVID-19 outbreak increased time spent at home, affecting lifestyle behaviors and individual action (7).

Considering eating habits, most of the participants did not face a big difference in the quantity and frequency of food intake before and during MCO (46.2% and 40.5%, respectively). On the contrary, a study carried out in Portugal (8) during the COVID-19 pandemic showed altered eating habits as there was a significant percentage of participants who reported that they started to eat in larger quantities (31.6%) more often (45.2%). The same result found in the study by Sidor et al. (6) from Poland stated that 43.5% of their participants had increased their food intake during the COVID-19 pandemic.

In United Arab Emirates (UAE), a study by Radwan et al. (12) reported that one of the unhealthy behavioral changes that persistently happened among the participants were increased food intake (32.0%). Besides that, our results showed that the majority of the subjects did not change the pattern of their number of meals during MCO. The majority of them declared eating three meals per day before and during the home confinement with percentages of 39.0 and 35.7%, respectively. However, a study in the MENA region showed an increase in the number of meals consumed per day, along with a reduction in the percentage of skipping meals by participants during the coronavirus pandemic (7).

During the COVID-19 pandemic, our study observed a positive change as most participants proclaimed an improvement in eating habits (eating patterns at home, reuse of surplus food, shopping list planning, menu planning, healthy food choices, and fresh food consumption) during MCO. It can also be observed that almost half of the participants (42.9%) did not consume soft drinks during home confinement. In addition, the majority of subjects also responded that they had eaten better during MCO (42.4%). On the contrary, a study by Scarmozzino et al. (13) in Italy found that the majority (52.9%) of their subjects claimed that there was an increase in the intake of comfort food (42.5%) such as chocolates, ice creams, desserts, and salty junk foods (23.5%) (13). In China, a study by Yang et al. (14) also reported that 38.2% of their subjects reported an increase in junk food consumption during the pandemic. The same result was observed from an online survey by Ammar et al. (15), which involved respondents from West Asia, North Africa, and Europe who reported that there was an elevated percentage of junk food and unhealthy food consumption during the COVID-19 pandemic.

Similarly, a study in Chile by Reyes-Olavarría et al. (9) reported that the majority (51.3%) of their respondents applied negative food habits with low intake of water and nuts, high consumption of fried food, and junk foods with low micronutrients, high sugar, fat, and sodium. Besides that, research by Di Renzo et al. (5) in Italy observed that 53.9% of the Italian participants had changed their eating habits. Particularly, there was an increase in food intake and consumption of unhealthy junk foods while staying at home during a pandemic. The study also stated that unhealthy eating patterns, such as unhealthy types of foods, uncontrolled eating behavior, frequent consumption of junk food between mealtimes, and quantity of main meals were all reported to have increased among the respondents (5). However, a study by Basu et al. (16) in India declared that 60.0% of their participants reported a decrease in fast food consumption during COVID-19.

In regards to supplement intake, the majority of the participants reported a hiher consumption of supplements (53.8%), with higher incidence among women (62.3%) compared to men (30.4%). López Moreno et al. (1), in Spain, also reported the same finding as 20.3% of their respondents, among which women (22.5%) more than men (15.3%), consumed supplements during the COVID-19 pandemic. This data were also similar to the study by Pérez-Rodrigo et al. (17), also in Spain, which reported that 21.3% of their participants, of which a higher percentage of women aged 35–54 years old, had been taking supplements during the COVID-19 outbreak.

In our results, the most frequent type of supplements taken by subjects was vitamin C, multivitamin, and fish oil. Similarly, Zaki et al. (18) also reported that the most common vitamin, mineral, and food supplements often consumed by Malaysian adults included Vitamin C, multivitamin, fish oil, and Royal jelly. This study also claimed that older-aged adult women with higher education levels and monthly income were more likely to consume dietary supplements (18). In Spain, a study by Pérez-Rodrigo et al. (17) also stated that multivitamins were the most frequent supplement taken by their participants, followed by the intake of vitamin D supplement (25.8%), and vitamin C (22%). In India, a study from Basu et al. (16) claimed that 35% of their respondents had been taking immunity boosters, of which the most popular choices were ginger and garlic, followed by multivitamins (15%) as a measure of immune protection.

As for tobacco, a small number of subjects (5.7%) in our study consumed tobacco during MCO. Nonetheless, in Australia, Stanton et al. (19) claimed that almost 93.0% of their respondents did not face any changes or decreased their smoking status since the beginning of the COVID-19 outbreak. A similar pattern could be observed from the study by Sidor et al. (6) in Poland, which reported that 40.0% of their subjects stated that their smoking habits did not change during the pandemic, while 14.8% of the subjects were not sure about their smoking habits. Meanwhile, a study by Tetik et al. (20) in Turkey claimed that 31.1% of their subjects had stopped smoking during the COVID-19 pandemic. A study by Klemperer et al. (21) in the United States also reported that 22.9% of their respondents lessened their smoking consumption, 21.2% decreased the usage of electronic cigarettes, and 16.2% stopped smoking during the COVID-19 pandemic.

However, a study by Malta et al. (22) in Brazil claimed that 34.0% of smokers among their subjects reported an increase in smoking habits during the pandemic, which was more frequent among adults aged between 18 and 29 years old. Twenty-two-point-five percent and 5.1% of their respondents reported an increase of 10 and 20 cigarettes in a day, respectively. Surprisingly, among the 22.5 percent who had an increment of 10 cigarettes a day, majority were women (28.9%; men, 16.8%). Increased smoking habits were reported to be one of the ways to cope with the stress faced by the participants during the COVID-19 outbreak (22). A study by Kolokotroni et al. (23) in Cyprus also reported an escalation in the trend of smoking habits. Particularly, 43.8% of their participants who were smokers had been smoking more frequently. The same goes for the study by López-Moreno et al. (1) in Spain, which observed that 7.5% of their participants had been smoking more often during COVID-19 pandemic (1, 23).

Regarding alcohol consumption, our study had observed that only a small number of subjects (7.6%) consumed alcohol during MCO. A corresponding result had been found by Sidor et al. (6) in Poland. In their study, 77.0% of their participants did not increase their alcohol consumption during the period of quarantine. However, 14.6% of the subjects reported an increase in alcohol consumption. This particular finding was more often found among alcohol addicts. Another 8.3% of the subjects were not sure about their alcohol consumption during the COVID-19 pandemic (6). Similarly, research by López-Moreno et al. (1) in Spain claimed that 18.3% of their subjects had taken alcohol more often during the COVID-19 outbreak compared to before the pandemic.

Reyes-Olavarría et al. (9) in Chile also observed that there was an increase in the alcohol consumption of their subjects (30.0%). This was consistent with the research by Australia's Foundation for Alcohol Research and Education which reported that 20.0% of Australians increased their purchase of alcohol at the beginning of COVID-19 outbreak and that 70.0% of Australians drank alcohol more than usual during COVID-19 pandemic (9, 19). In our study, beer, red wine, and traditional rice wine were the alcoholic beverages most consumed by drinkers among the participants. The same result could be observed from a study by Pérez-Rodrigo et al. (17) in Spain in which wine and beer were frequently consumed (68.5%).

Regarding sleeping patterns, our study observed that the average hours of sleep by government servants during MCO was 6.67 ± 1.353 h per day, higher than 6.30 ± 1.146 h before MCO. This showed a statistical significance between sleep hours prior and during the home confinement. Besides that, the majority of the subjects (53.8%) in our study also stated that their sleep quality did not change during the pandemic. There was also an increase in the total number of subjects with more than 7 h of sleep during home confinement (49.5%) compared to prior home confinement (31.4%). Equivalently, the study by Di Renzo et al. (5) reported that the sleeping time among the subjects had changed during the home confinement period in Italy. Another research in Italy by Cellini et al. (24) also claimed that there was a change in sleeping patterns of the respondents as the percentage of respondents that lack sleep increased fromv 40.5% before the pandemic to 52.4% during the pandemic.

Cheikh Ismail et al. (7) also observed that the percentage of subjects who reported poor sleep quality had increased from 17.1% before the COVID-19 outbreak to 29.2% during the COVID-19 pandemic. The percentage of participants who reported experiencing sleep disturbances during the pandemic had increased (63.2%) compared to the percentage before the pandemic (53.1%) (7). On the contrary, a study by Antunes et al. (8) in Portugal stated that the majority of the respondents did not face any changes in their sleeping patterns. In addition, Stanton et al. (19) claimed an inconsistent result from their research as half of the respondents (50.7%) reported no sleep quality changes since the COVID-19 outbreak. Still, another 40.7% of the respondents stated an adverse difference in their sleep quality.

The COVID-19 pandemic put pressure on some individuals as they had to restrict their social activities (25). Self-adaptation to sitting at home for an extended period during a pandemic contributed to a higher stress level (26). According to Di Renzo et al. (5), factors of stress included uncertainty about the quarantine period, fear of the possibility of being infected, banned on going to the hospital unless for necessary scenarios, disappointment, boredom, uncertainty with the future, fear of large financial losses, and long-term effects that would lead toward emotional eating habits.

During the period of MCO, our study had perceived that 25.7% of participants (17.9% men and 28.5% women) reported nervousness and distress to a greater extent. In addition, Di Renzo et al. (5) stated that almost half of the respondents in Italy reported anxious feelings toward their eating habits, among which which the percentage of anxiety among women was higher than men. In the UAE, a study by Radwan et al. (12) observed that the majority of their participants reported high-stress levels and anger during COVID-19.

Social isolation, fear of COVID-19, anxiety, loneliness, and boredom had been shown to influence eating behaviors (4). All participants in our study agreed that there was a relationship between food and health. The majority (56.2%) of the subjects (53.6% men and 57.1% women) stated that their mood positively affected their eating habits during home confinement. In contrast, a study by López-Moreno et al. (1) in Spain reported that 54.4% of the respondents experienced negative changes in food habits during the isolation period. This included an increase in food intake and consumption of comfort food as it was reported to reduce the emotional problems or stress of the participants (1). Flaudias et al. (27) also stated that higher levels of stress and anxiety during isolation among their participants were related to overeating.

Based on Ammar et al. (15), the increase in consumption of junk food and unhealthy food was caused by anxiety factors, boredom, or depression experienced by the subjects during the pandemic outbreak. A study by de Faria Coelho-Ravagnani et al. (28) also claimed that the negative psychological effects had caused emotional nutrition and triggered a craving of sweetened foods. Radwan et al. (12) from the UAE reported that the increase in food intake among the subjects was caused by the fear and anxiety that they felt during the pandemic outbreak. On the other hand, Basu et al. (16) from India reported a decrease in the consumption of fast food that was related to the strict restrictions made by the Indian government by banning the Indian citizens from going out of their houses. Hence, respondents might have been scared of ordering food through online delivery apps as they were scared of the spread of COVID-19 (16).

From our study, most of the participants (49.1%; 57.2% men and 46.1% women) were classified as emotional eaters or very emotional eaters. Meanwhile, Di Renzo et al. (5) stated that women from the study were more prone to emotional eating behaviors. Particularly, they had increased their food intake in order for them to feel better or used food in response to the anxiety that they felt during COVID-19 outbreak (5). Apart from that, 36.7% of participants from our study (26.8% men and 40.3% women) were categorized as low-emotional eaters, while 14.3% of participants (16.1% men and 13.6% of women) were classified as non-emotional eaters, respectively. Our study had observed that the classification of emotional eater was significantly correlated with the quantity of food intake and frequency of mealtime.

Regarding supplement intake, our study reported that there was no relationship between supplement intakes and emotional levels during MCO. Di Renzo et al. (5) claimed that stress, restlessness, anxiety, and insomnia had caused a significant increase in the usage of medicines and additional foods among women compared to men. Besides that, the study also stated that women had frequent and higher psychological responses toward pressure than men (5). A study in China by Zhoa et al. (29) concluded that supplement intake was due to how the participants were more concerned about nutrition and health condition during the COVID-19 outbreak.

As for smoking habits, our study observed only 5.6% of non-emotional and low emotional eaters, while 6.4% of emotional and very emotional eaters had been smoking tobacco during MCO. This was in line with the results of Tetik et al. (20) in Turkey that claimed that a significant percentage of their subjects had stopped smoking during the COVID-19 pandemi. They related this result with the participant's perceptions on how smoking during the pandemic outbreak would have a negative effect on one's health (20). Similarly, Klemperer et al. (21) also reported that a significant number of their total subjects lessened their smoking consumption, decreased the usage of electronic cigarettes, and/or stopped smoking during the COVID-19 pandemic due to the subjects' feeling of fear and concern to take better care of their health status during a pandemic outbreak.

Clay et al. (30) had mentioned that stress had been claimed as the prominent risk factor in the onset and maintenance of alcohol misuse. However, our study reported that only 6.8% of emotional and very emotional eaters had consumed alcohol during MCO. This result was against the findings from López-Moreno et al. (1) in Spain, which claimed that some of their subjects had taken alcohol more often during the COVID-19 outbreak compared to before the pandemic. Eventually, the study concluded that the increase of alcohol consumption was due to pandemic situation which led to the increased stress levels of some individuals (1). Besides that, almost 30.0% of Australian adults also consumed more alcohol to overcome the psychological problems faced during the pandemic (19).

Pérez-Rodrigo et al. (17) reported that stress was the major factor in the onset of alcohol abuse during the COVID-19 pandemic. The pandemic also provoked or exacerbated individual psychological problems, especially when receiving alarming information from news and social media related to COVID-19 (31). In addition, Stanton et al. (19) stated that negative changes in alcohol consumption among Australians were due to factors of depression, anxiety, and stress during MCO. Chodkiewicz et al. (32) also claimed that individuals who regularly consumed alcohol were less likely to find something positive and unable to adapt to the challenging situation of a pandemic outbreak.

Regarding sleeping habits, our study observed that 43.8% of the participants (37.5% men and 46.1% women) reported not having any difficulty falling asleep. There was also no relationship between quality of sleep and emotional level among participants during MCO. In contrast, research by Cellini et al. (24) in Italy reported that the score of above five in the Pittsburg Sleep Quality Index (PSQI) had increased among the participants (from 40.5% before pandemic to 52.4% during the COVID-19 pandemic), indicating sleep deprivation. The study also reported that changes in sleep quality (global PSQI >5) were higher in participants with a DASS-21, indicating that participants experienced symptoms of depression and anxiety. Therefore, the researchers noted a higher decline in sleep quality in people with high levels of depression, anxiety, and stress, which carried the same conclusion as a study by Stanton et al. in Australia (19, 24).

A study in MENA also claimed of increasing percentages of participants who reported poor sleep quality and sleep disturbances during the COVID-19 pandemic. It concluded that there was an increased percentage of participants who reported feeling lazy and less energetic during the pandemic period (7). Altena et al. (33) also reported that the effects on lifestyle changes, travel restrictions by the government, increased depression, anxiety, and stress associated with the COVID-19 pandemic might have significant negative effects on individuals' sleep habits and sleep quality.

The importance of emphasizing participation in physical activity in this pandemic period is indicated to attain a healthier life. The quarantine period was related to stress/depression, leading to unhealthy diet and reduced physical activity (34). Based on our study, most participants reported engaging in physical activity during MCO. This was categorized into four aspects, namely, continuing exercise with the same training patterns, performing exercise but with lower intensity, performing exercise but with a shorter period, and only started doing exercise during home confinement.

Similarly, a study by Cancello et al. (35) also reported that 32% of the participants who exercised actively before the MCO claimed to continue and increase their physical activity during a pandemic outbreak. On the other hand, 27% of the inactive participants before the MCO had reported starting their exercise routine during the pandemic period (35).

However, our findings seemed to contradict previous studies by López-Moreno et al. (1), Yang et al. (14), and Husain et al. (36). who reported that most of their participants did not practice physical exercise during home confinement due to the closure of all sports centers and recreation areas. Thus, the facilities and opportunities for performing physical activity were minimal.

Our result also observed that 132 subjects reported a mean value of total MET of 3495.8 (SD 3862.7) min/week. Almost half of the subjects were categorized with high MET (45.5%), of which 59.0% were men and 39.8% were women. Most subjects with non-emotional and low emotional eaters had high MET (50.0%) during MCO. The same finding was reported by Antunes et al. (8), who claimed that most of the participants engaged in performing regular physical activity. Among which, 49.6% were classified as moderate level and 18.5% were classified as high level according to the category in IPAQ (8).

An interesting finding on changes of body weight before and during MCO was observed in our study. Particularly, the mean values of body weight and BMI among subjects were reported of being decreased during MCO compared to prior, which might have been caused by the increased effort spent on doing physical activity and improvement in eating habits. On the other hand, previous studies have reported that there was an increase in body weight among their participants. In MENA, about one-third of the surveyed individuals (30.3%) reported weight gain during coronavirus pandemic. This finding further explained that the weight gain that occurred was not gained with the subsequent engagement in any physical activity during the coronavirus pandemic (7). Similarly, López-Moreno et al. (1) reported that 38.8% of surveyed individuals in Spain increased their body weight by an average of 2.6 kg during the lockdown. The previous study in Chile reported that negative food habits were related to the negative changes in weight among their respondents (9).

### Strength and Limitations

This cross-sectional study on lifestyle, psychological factors affecting eating habits, and physical activity of the government servants during pandemic COVID-19 was the first to be conducted in Malaysia. As this study was conducted online, it appeared to have some limitations. Firstly, the data for weight and level of physical activity were self-reported, which could lead to the possibility of having under-reported and over-reported data for both categories. However, to minimize this issue, detailed instructions were written in the questionnaire prior to questions on anthropometry. However, the López-Moreno et al. (1) questionnaire that were used was not validated for the Malaysian population. In addition, the answers were not classified as a scoring system. Therefore, each of the components studied could not be categorized. Thus, less statistical analysis could be done to test various research hypotheses toward the result obtained from the study. Lastly, it was also found that a higher proportion of women responded to the survey compared to men, which is consistent in other similar health surveys (1, 5, 17).

## Conclusion

As an overview of our results, an interesting finding of this study was that positive changes could be seen in lifestyle behaviors, weight changes, and physical activity during MCO as most participants reported having improvements in their eating behavior. Particularly, in the aspects of the following: their eating patterns at home, reuse of surplus food, shopping list planning, menu planning, healthy food choices, fresh food consumption, and less intake of soft drink. Apart from that, some participants also responded that they had been eating better during MCO and decreased their body weight and BMI. Finally, most participants also claimed that they performed physical exercise. However, some unfavorable behaviors were reported, such as an increase in deep frying for cooking methods, and the majority of the respondents were classified as emotional eater or very emotional eater in this study.

During this period of a MCO, it is crucial to understand government servants' lifestyle behaviors and study the psychological dimensions as these play a significant role in the quality of life of government servants. Such information will help in designing better strategies in the future to improve the economic and health status among government servants in Malaysia while the MCO is still being implemented.

## Data Availability Statement

The raw data supporting the conclusions of this article will be made available by the authors, without undue reservation.

## Ethics Statement

The studies involving human participants were reviewed and approved by Research and Ethics Committee of The National University of Malaysia (UKM) with ethical code: UKM PPI/111/8/JEP-2021-269. The patients/participants provided their written informed consent to participate in this study.

## Author Contributions

NH, MS, and NA directed the statistical analyses and reviewed and edited the manuscript. ZG, SA, and NA were responsible for data collection, conducted statistical analysis, and wrote the initial draft of paper. NH conceived the idea, data collection, and coordinated all steps of this project. All authors participated in the project design. All authors contributed to the article and approved the submitted version.

## Conflict of Interest

The authors declare that the research was conducted in the absence of any commercial or financial relationships that could be construed as a potential conflict of interest.

## Publisher's Note

All claims expressed in this article are solely those of the authors and do not necessarily represent those of their affiliated organizations, or those of the publisher, the editors and the reviewers. Any product that may be evaluated in this article, or claim that may be made by its manufacturer, is not guaranteed or endorsed by the publisher.
